# Pore Filling Effect of Forced Carbonation Reactions Using Carbon Dioxide Nanobubbles

**DOI:** 10.3390/ma13194343

**Published:** 2020-09-29

**Authors:** Jihoon Kim, Ryoma Kitagaki, Heesup Choi

**Affiliations:** 1 Department of Civil Engineering and Architecture, Muroran Institute of Technology, Hokkaido 0508585, Japan; bmjhun@mmm.muroran-it.ac.jp; 2 Division of Human Environmental System, Hokkaido University, Hokkaido 0600808, Japan; 3 Department of Civil and Environmental Engineering, Kitami Institute of Technology, Hokkaido 0908507, Japan

**Keywords:** cementitious materials, nanosized ultrafine CO_2_ bubble, CaCO_3_, forced carbonation, surface modification

## Abstract

Various methods for repairing and modifying concrete surfaces have been proposed and applied to improve the durability of existing concrete structures. Surface modification through forced carbonation is a method of densification that forms calcium carbonate in the pores on the surface of concrete to improve its durability. In this study, to evaluate the applicability of this surface modification method to existing buildings, a series of experiments was conducted in which mortar specimens were repeatedly immersed in a carbon dioxide nanobubble aqueous solution. By evaluating the weight change and absorption rate, it was determined that the higher the water/cement ratio of the mortar specimen, the higher the pore filling effect owing to immersion in the carbon dioxide nanobubble aqueous solution. In addition, the effect of clogged pores generated by the precipitation of calcium carbonate was confirmed, and it was found that the higher the water/cement ratio of the mortar specimen, the higher the pore filling effect due to clogging. We believe that our findings contribute to the development of research and construction practices associated with concrete repair and restoration.

## 1. Introduction

From the standpoint of environmental load and resource utilization issues, the long-term prolongation of the service life of concrete structures has become an essential element in the construction industry. In recent years, rather than continuously building new buildings following a scrap and build approach, it has become more common to respond to changes in building needs by renovating existing facilities through innovation and conversion [1]. As a result, it is often necessary to extend the service life of concrete structures, and the most important factor in doing so is typically the concrete surface durability [2]. Problems related to the durability of concrete structures begin with the surface penetration of deterioration factors such as carbonate ions, chloride ions, and water [3]. To improve the durability of concrete structures, research has been conducted on a technique to increase concrete volume by carbonating the calcium in the concrete, such as in calcium hydroxide (Ca(OH)_2_), with the carbon dioxide (CO_2_) in the atmosphere, creating calcium carbonate (CaCO_3_) that fills the pores in the concrete to maintain a dense surface [4,5,6,7,8,9,10,11,12,13,14,15]. On concrete surfaces where cracks exist, self-healing is observed, which partially fills in cracks due to rehydration of cement particles and CaCO_3_ precipitation [13,14]. In addition, in the concrete self-healing mechanism by the reaction of Ca^2+^ in concrete and CO_2_ dissolved in water, the generated CaCO_3_ is not easily dissolved in water [15]. This type of treatment can be expected to slow the penetration of deterioration factors into the concrete element through the densified concrete surface. However, to realize a densified concrete surface through intentional atmospheric carbonation, it is necessary to conduct the reaction in a controlled chamber; thus, at this time, carbonation treatment can only be used on precast concrete and is difficult to apply to existing concrete structures [5,6,7,8,9,10,11,12].

Therefore, in order to develop a method of densifying the surface of existing concrete structures in the field through a forced carbonation reaction, this study examines the effect of applying carbon dioxide nanobubble water to a concrete surface. These nanobubbles have a small bubble diameter, a slow rise rate in water, contain high pressure, and their surface is negatively charged [16,17,18]. The small bubble diameter is directly related to the high pressure inside the bubble, thereby increasing the partial pressure of the gas and increasing its solubility in water. In addition, in the pore filling effect, CaCO_3_ having a uniform morphology and crystal structure is required. These properties of CaCO_3_ play an important role in the pore filling effect. The form of CaCO_3_ depends on the solution pH, feed rate of reactants, and solution composition [19,20,21,22]. Previously, Onoe et al. [23] examined changes in pH when carbon dioxide microbubbles and carbon dioxide millibubbles were each supplied to ion-exchanged water at the same rate. It was reported that carbon dioxide supplied in microbubbles with small diameters (30–70 nm) exhibited a greater degree of dissolution than that supplied in millibubbles. Thus, given the promising results of previous microbubble applications, the purpose of this study is to evaluate the filling effect of repeated CO_2_ nanobubble water impregnation on the surface density of mortar specimens.

## 2. Materials and Methods

### 2.1. Materials and Specimen Preparation

Mortar specimens were used to measure the effect of carbonation by repeated impregnation of CO_2_ nanobubble water. Because the CO_2_ nanobubbles react with the cement paste component, the volume ratio of the aggregate and the cement paste in the mortar were set to be constant. Mortar specimens with water/cement ratios (W/C) of 0.4, 0.5, and 0.6 were prepared for a unit volume as shown in Table 1. The cement used was ordinary Portland cement (density = 3.16 g/cm^3^) (Taiheiyo Cement, Tokyo, Japan), and the fine aggregate was a water-based land sand (absolute dry density = 2.55 g/cm^3^) (Abbey Road Co., Ltd, Shiraoi, Japan). Fine aggregate was controlled to dry surface condition according to Japanese Industrial Standards (JIS A 1109) [24]. After mixing, each mixture was kneaded repeatedly until the bleed water was exhausted and then gently poured into a 10 mm diameter, 100 mm long polypropylene resin form to minimize air entrainment. Each mixture was sealed and cured at 20 °C for one day after the start of mixing. Thereafter, the mortar specimens were demolded and cured in water at 60 °C for seven days. Then, after being returned to 20 °C, the mortar specimens were cut into small pieces that were approximately 15 mm long. These pieces were next immersed in acetone for 6 h to stop the hydration reaction, and then kept in a vacuum desiccator for 24 h to confirm that the measured weight loss had converged to zero.

### 2.2. Immersion Prodecure and Measurements

We prepared five 50 mL glass bottles for each W/C ratio to evaluate the effects of 0, 1, 2, 3, and 4 repeated immersions in CO_2_ nanobubble water, making for a total of 15 tests. Here, we repeated immersion up to 4 times to examine the efficiency when inducing the carbonation reaction repeatedly. Three pieces of each mortar specimen were placed in each beaker to form a sample set. The total absolute dry weight of the samples placed in each bottle was measured using an electronic balance with an accuracy of 0.01 g; this value was taken as the initial absolute dry weight $W_{d}$ [g]. Once a day, the mortar samples were subjected to the immersion procedures described in Table 2. The natural impregnation method consists of placing a predetermined volume of acetone and Ca(OH)_2_ saturated aqueous solution into the bottle containing a sample set and leaving it for a predetermined period of time without any operation to allow the solution to penetrate the specimens. The ultrasonic impregnation method is similar to the natural impregnation method, except that the immersed sample is subjected to an ultrasonic cleaner for a predetermined period of time during the impregnation process. The ultrasonic impregnation method has been previously used to aid solution infiltration into the internal voids of many porous materials [25].

The immersion times applied in this study were set in preliminary experiments by confirming the time required for water to impregnate the specimen to its center. The volume of the solution used in each procedure was then set to be about 50 times the amount of water absorbed by the specimen. It should be noted that before each operation, the solution used in the previous operation was discarded. The specimens were immersed in a nanobubble solution created by adding CO_2_ nanobubble water to 30 L of ion-exchanged water at a flow rate of 1 L/min for 20 min using a mechanical swirling-type nanobubble generator, creating a solution with a pH of 4.5. The hydration reaction was repeatedly stopped during the immersion procedure detailed in Table 3 through a combination of natural and ultrasonic immersion in acetone. Moisture was repeatedly removed by vacuum desiccator; the time required to do so was determined from preliminary tests based on the time after which the weight loss converged to zero.

**Figure 1:**
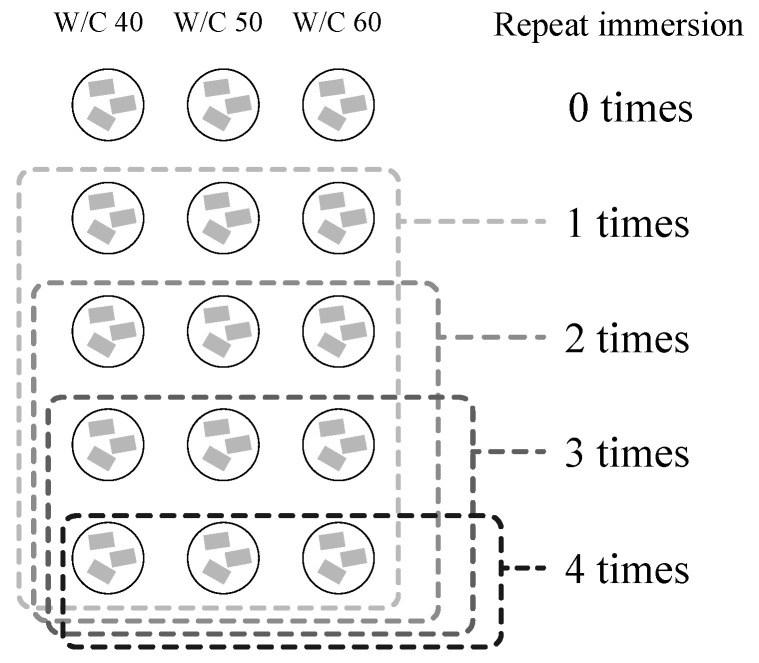
Repeated mortar specimen immersion method.

The number of repeated immersions for each specimen set was accomplished by reducing the number of bottles tested at each W/C ratio, as shown in Figure 1 [26]. After all CO_2_ nanobubble water immersion procedures had been completed, the following mortar sample set weights were measured using an electronic balance with an accuracy of 0.01 g: $W_{d}$ [g], the absolute dry weight of the specimens after drying in vacuum desiccator for 1 day, $W_{s}$ [g], the surface dry weight of the specimens after immersion in ion-exchanged water for 1 day after drying, and $W_{w}$ [g], the submerged unit weight of the specimens after immersion in ion-exchanged water for 1 day after drying.

### 2.3. Microscopic Investigations

In order to microscopically investigate the filling effect of CaCO_3_ by CO_2_ nanobubble water in these experiments, the concentration of Ca(OH)_2_ at the age before immersion produced through the hydration reaction was calculated based on Papadakis as follows [27,28,29]:

(1) $$\left[\mathrm{Ca}{\left(\mathrm{OH}\right)}_{2}\right]=\frac{3}{2}{\left[C_{3}S\right]}_{0}F_{C_{3}S}+\frac{1}{2}{\left[C_{2}S\right]}_{0}F_{C_{2}S}-4{\left[C_{4}\mathrm{AF}\right]}_{0}F_{C_{4}\mathrm{AF}}-{\left[C_{3}A\right]}_{0}F_{C_{3}A}+{\left[{C\bar{S}H}_{2}\right]}_{0}$$

(2) $$F_{i}\left(t\right)=1-\left[i\right]/{\left[i\right]}_{0}=1-{\left(1-k_{H,i}t\left(1-n_{i}\right)\right)}^{1/\left(1-n_{i}\right)}$$

where $F_{i}\left(t\right)$ is reaction fraction of substance *i* at time *t* [-]; [i]  and ${\left[i\right]}_{0}$ is concentration of substance *i* at time *t* and the initial concentration, respectively [mol/m^3^]; $k_{H,i}$ is reaction rate coefficient of substance *i* at 20 °C [1/s]; $n_{i}$ is a constant obtained by fitting [-].

In Equation (2), the reaction rate coefficient k was calculated using the two temperatures applied in this study, 20 °C and 60 °C, according to Arrhenius using [30]:

(3) $$k=\chi \exp\left(-\frac{E_{a}}{RT}\right)$$

where $E_{a}$ is activation energy [J/mol]; R is gas constant [J⋅K^−1^⋅mol^−1^]; T is absolute temperature [K]; χ is proportional constant [-].

The porosity ϵ(t) due to hydration reaction and carbonation can be obtained by

(4) $$\epsilon \left(t\right)=\epsilon_{0}-\Delta \epsilon_{H}\left(t\right)-\Delta \epsilon_{c}$$

where $\epsilon_{0}$ is initial porosity in fresh concrete [-]; $\Delta \epsilon_{H}\left(t\right)$ is decrease in porosity due to hydration reaction [-]; $\Delta \epsilon_{c}$ is reduction rate of porosity due to carbonation reaction [-].

(5) $$\epsilon_{0}=\frac{\frac{w}{c}\;\frac{\rho_{c}}{\rho_{w}}\left(1-\epsilon_{air}\right)}{1+\frac{w}{c}\;\frac{\rho_{c}}{\rho_{w}}+\frac{a}{c}\;\frac{\rho_{c}}{\rho_{a}}}+\epsilon_{air}$$

where $\frac{w}{c}$ is water-cement ratio [-]; $\frac{a}{c}$ is aggregate-cement ratio [-]; $\epsilon_{air}$ is volume fraction of concrete in entrapped or entrained air $\epsilon_{air}$ [-]; $\rho_{c}$ is density of cement [kg/m^3^]; $\rho_{a}$ is density of aggregate [kg/m^3^]; $\rho_{w}$ is density of water [kg/m^3^].

## 3. Results and Discussion

### 3.1. Rate of Weight Increase, Absolute Dry Density, and Rate of Water Absorption

**Figure 2:**
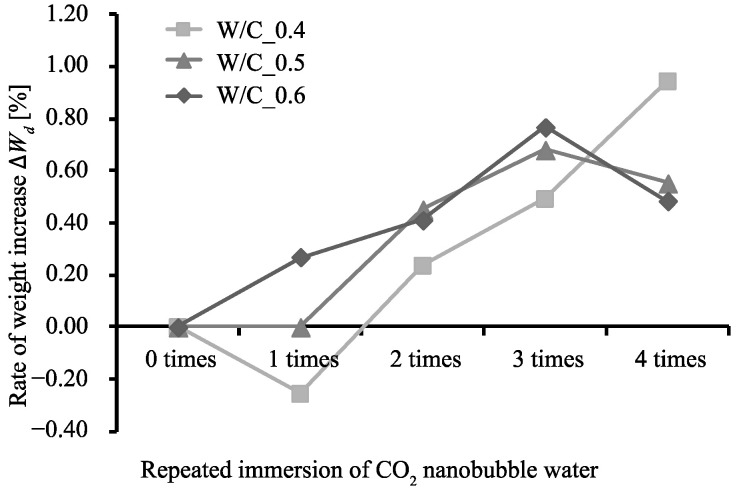
Relationship between number of immersions and rate of specimen weight increase.

**Figure 3:**
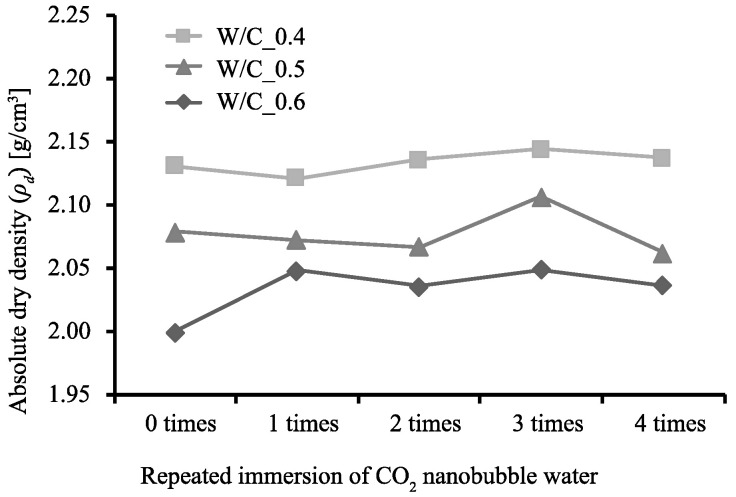
Relationship between number of immersions and absolute dry density.

**Figure 4:**
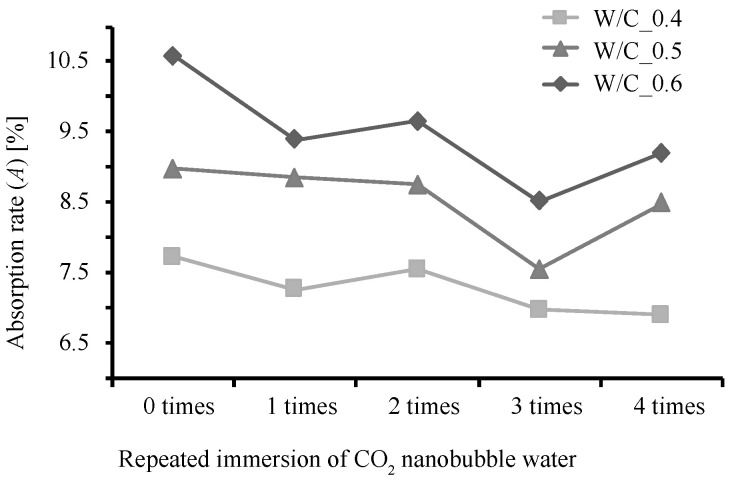
Relationship between number of immersions and rate of water absorption.

Figure 2, Figure 3 and Figure 4 show the rate of weight increase ($\Delta W_{d}\;\left[\%\right]$), absolute dry density ($\rho_{d}\;\left[g/{\mathrm{cm}}^{3}\right]$), and rate of water absorption (A [%]) for each W/C ratio and number of immersions, as obtained from the measurements conducted in this study.

(6) $$\Delta W_{d}=\frac{W_{d}-W_{d}{}^{0}}{W_{d}{}^{0}}\times 100$$

(7) $$\rho_{d}=\frac{W_{d}}{\left(W_{s}-W_{w}\right)∕\rho_{w}}$$

(8) $$A=\frac{W_{s}-W_{d}}{W_{d}}\times 100$$

where $W_{d}{}^{0}$ is absolute dry weight before immersion [g].

**Figure 5:**
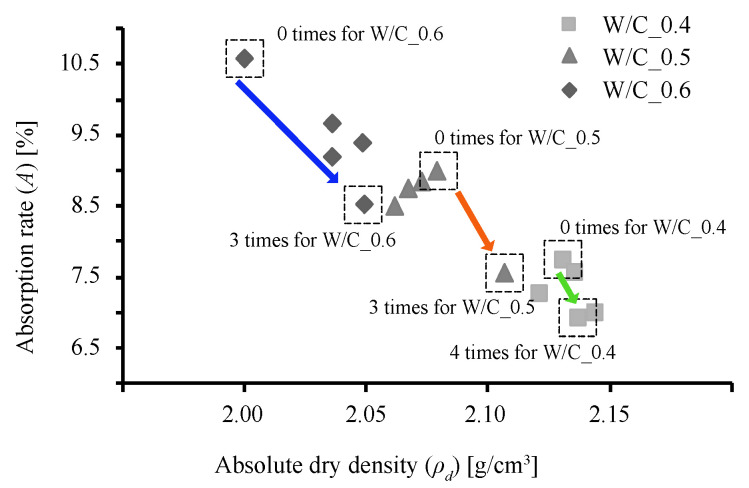
Relationship between absolute dry density and rate of water absorption.

It can be seen that as the number of immersions increases, so does the value of $\Delta W_{d}$. Specimen W/C_0.4 exhibits the largest $\Delta W_{d}$ after being subjected to the immersion procedure 4 times. However, specimens W/C_0.5 and W/C_0.6 exhibit their maximum $\Delta W_{d}$ values after being subjected to the immersion procedure 3 times, after which the $\Delta W_{d}$ values dropped. This is considered to be due to the consumption of Ca(OH)_2_ during the third immersion, after which the C-S-H gel was decomposed and eluted during the fourth immersion. [31] The values of $\rho_{d}$ however, show little change regardless of the number of times the specimens were subjected to the immersion procedure. On the other hand, the A values are at their minimums after 3 immersions, the same number of immersions required for the rate of weight increase to reach its maximum. Generally, the higher the density, the lower the water absorption; this relationship indicates that as the Ca(OH)_2_ is changed to CaCO_3_, the weight of the specimen increases, and this increase in volume has a pore filling effect. This tendency can be seen in the relationship between $\rho_{d}$ and A in Figure 5, in which the absorption rate of the W/C_0.6 mortar after immersion decreases to the absorption rate of the W/C_0.5 mortar before immersion, and the absorption rate of W/C_0.5 mortar after immersion decreases to the absorption rate of the W/C_0.4 mortar before immersion.

### 3.2. Pore Filling Effect of CO_2_ Nanobubble Water

The porosity P [%] and the porosity reduction rate  ΔP [%], an index representing the reduction in minimum porosity with repeated immersion, defined relative to the porosity of a test specimen that has not been impregnated with CO_2_ nanobubble water can be respectively obtained using the measured water absorption results as follows:

(9) $$P=\frac{\left(W_{s}-W_{d}\right)∕\rho_{w}}{\left(W_{s}-W_{w}\right)∕\rho_{w}}\times 100$$

(10) $$\Delta P=\frac{P_{0}-P_{min}}{P_{0}}\times 100$$

where $P_{0}$ is porosity before any immersion [%]; $P_{min}$ is smallest porosity after immersion for four times [%].

Note that when the Ca(OH)_2_ in a specimen, which weighs 74 g/mol, is changed to CaCO_3_, which weighs 100 g/mol, the overall weight of the specimen increases by 26 g/mol. Based on the Papadakis model described in Section 2.3, the weight increase $\;w_{max}\;$ [g] and the reaction rate r  [%] when the Ca(OH)_2_ is completely carbonated are respectively calculated by

(11) $$w_{max}=\left(100-74\right)\cdot \left[\mathrm{Ca}{\left(\mathrm{OH}\right)}_{2}\right]\cdot V$$

(12) $$r=\frac{W_{d}-W_{d}{}^{0}}{w_{max}}\times 100$$

The value of $\Delta \epsilon_{c}$ in Equation (4) represents the decrease in porosity when the Ca(OH)_2_ is completely carbonated, and is calculated using the final concentrations of Ca(OH)_2_ and C-S-H gel. However, not all Ca(OH)_2_ and C-S-H gels are carbonated in this experiment. In consideration of the reaction rate obtained by Equation (12), the void reduction ratio Δϵ [%] resulting from carbonation was calculated based on that determined from the change in specimen weight; that is, the Δϵ was calculated from the volume of precipitated CaCO_3_ determined as follows:

(13) $$\Delta \epsilon =\frac{\Delta \epsilon_{c}\cdot \left(\frac{r}{100}\right)}{\epsilon_{0}-\Delta \epsilon_{H}\left(t\right)}\times 100$$

**Figure 6:**
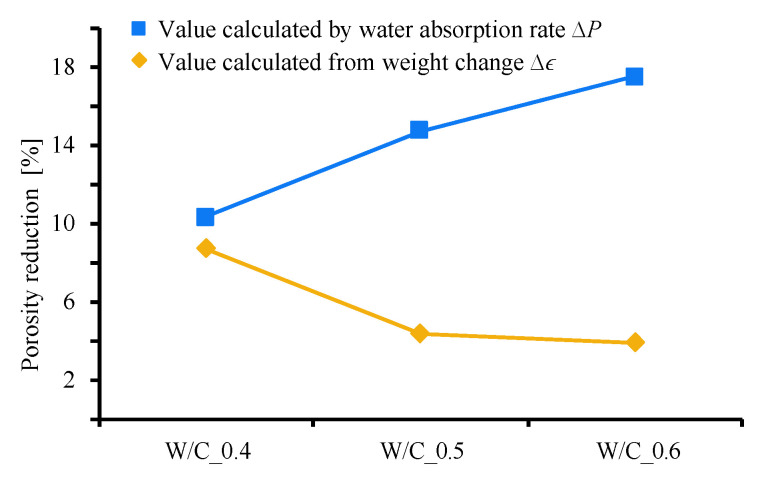
Comparison of porosity reduction ratios calculated two ways.

Figure 6 shows a comparison of the Δϵ obtained using the change in weight and the ΔP obtained using the water absorption rate. Clearly, the ΔP calculated using the water absorption rate increases as the W/C ratio increases. This indicates that specimens with a larger W/C ratio—that is, a larger original porosity—exhibit a larger ΔP. For this result, the influence of external supplied Ca(OH)_2_ by ‘saturated Ca(OH)_2_’ used in the precipitation process can also be considered. In this process, there is a possibility that relatively more external Ca(OH)_2_ penetrates into the specimen with high W/C.

On the other hand, the Δϵ calculated using the change in weight decreases as the W/C ratio increases. From these results, the CO_2_ nanobubble immersion method can actually expect a better absorption rate reduction effect than the absorption rate reduction effect by pore filling due to the production of CaCO_3_. Likewise, this result may be influenced by Ca(OH)_2_ supplied from the outside. Therefore, we performed preliminary experiments and simulations, but the effect was not large, and the trend did not change.

**Figure 7:**
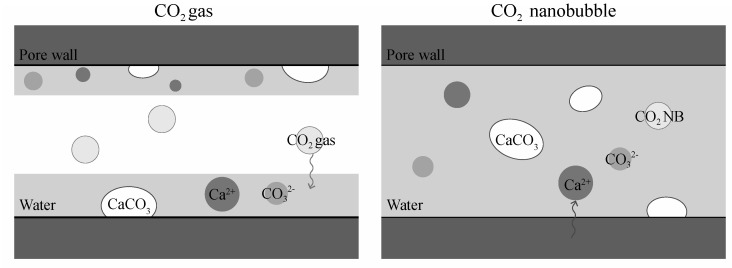
Schematic diagram of carbonation with CO_2_ gas (**left**) and CO_2_ nanobubble water (**right**).

As shown in the conceptual diagram of Figure 7, in an ordinary carbonation reaction, CO_2_ gas is supplied to the pores from the outside via the gas phase, and then dissolved in the liquid phase to become carbonate ions, which react with calcium ions to form CaCO_3_ on the inner surfaces of the pores. On the other hand, in a carbonation reaction using CO_2_ nanobubble water, the pores are filled with the CO_2_ nanobubble water, and the CaCO_3_ precipitation reaction then occurs in various places in the pores. Additionally, because the precipitation rate of CaCO_3_ is much faster in CO_2_ nanobubble water than in CO_2_ gas, the likelihood of the pores rapidly closing is increased. Such a rapid pore closing effect causes the void reduction ratio obtained using the water absorption rate to be greater than the void reduction ratio obtained using the change in weight. Section 3.3 accordingly evaluates the tendency of this clogging effect in each W/C ratio specimen.

### 3.3. Quantification of Pore Closing Effect by CO_2_ Nanobubbles

To examine the effect of clogging when the carbonation reaction is performed using CO_2_ nanobubble water, two types of pore filling are evaluated: one with no clogging and one with clogging. First, the water absorption weight *A* [-] and the water absorption volume *A*′ [-] obtained from the experiments are related by

(14) $$A=\frac{\rho_{w}}{\rho_{c}}\frac{v}{V}=\frac{\rho_{w}}{\rho_{c}}A^{\prime }$$

where V is total volume [cm^3^]; v is pore volume [cm^3^].

The Ca(OH)_2_ is converted to CaCO_3_ by the carbonation reaction, and the increased volume of CaCO_3_ is defined as ΔV. At this time, it is assumed that no cavity exists in ΔV. Considering that ΔV depends on the carbonation reaction rate *R*, the following equation holds:

(15) $$\Delta V=\Delta {\bar{V}}_{CH}\times \left[\mathrm{Ca}{\left(\mathrm{OH}\right)}_{2}\right]\times V_{0}\times \frac{R}{100}$$

where $\Delta {\bar{V}}_{CH}$ is increase in volume per mole of Ca(OH)_2_ used for carbonation [cm^3^]. Here, in applying the theoretical model of the porosity and the amount of hydration product produced by Papadakis [27], it was assumed that only Ca(OH)_2_ contributed to the void change due to carbonation in the specimen subjected to forced carbonation using CO_2_ nanobubbles.

If the volumetric water absorption that occurs after the carbonation reaction is defined as *A_non_*′ [-] and the initial water absorption is defined as *A*_0_*′* [-], then *A_non_′* [-] can be expressed as

(16) $${A_{non}}^{\prime }=\frac{{A_{0}}^{\prime }V_{0}-\Delta V}{V_{0}}={A_{0}}^{\prime }-\frac{\Delta V}{V_{0}}$$

**Figure 8:**
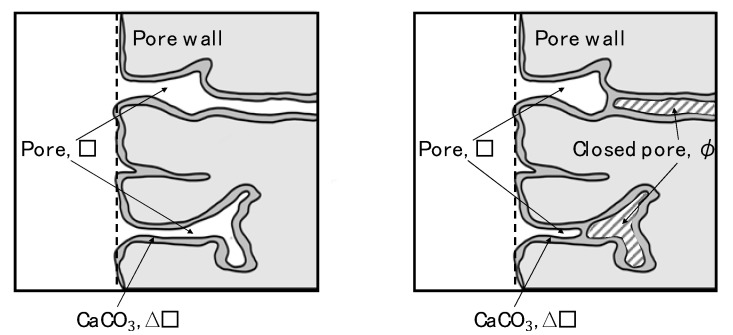
Schematic diagram of the state of pores without (**left**) and with (**right**) clogging effect.

Figure 8 is a schematic diagram of a pore without and with the clogging effect. In the case of the clogged pore, R is also equal to r, and thus the volume increased by carbonation is ΔV. However, unlike the unclogged pore, the ΔV of the clogged pore includes the cavity trapped behind the clog formed by precipitated CaCO_3_. Here, pores communicating with the outside air are called open pores, and pores not communicating with the outside air are called closed pores. The volumetric water absorption (${A_{s}}^{\prime }$) and closed pore volume (φ) generated by clogging after carbonation can be respectively expressed as

(17) $${A_{s}}^{\prime }=\frac{{A_{o}}^{\prime }V_{0}-\Delta V-\varphi }{V_{0}}={A_{non}}^{\prime }-\frac{\varphi }{V_{0}}$$

(18) $$\varphi =\left({A_{non}}^{\prime }-{A_{s}}^{\prime }\right)V_{0}$$

The reported decrease in void volume due to the clogging effect is thus actually a decrease in the apparent void volume as opposed to the decrease in actual void volume due to the increasing volume of CaCO_3_ formation. Therefore, using φ and ΔV, the closed pore volume increase rate per unit volume increase due to the generation of CaCO_3_ (S) can be expressed as

(19) $$S=\frac{\varphi }{\Delta V}$$

**Figure 9:**
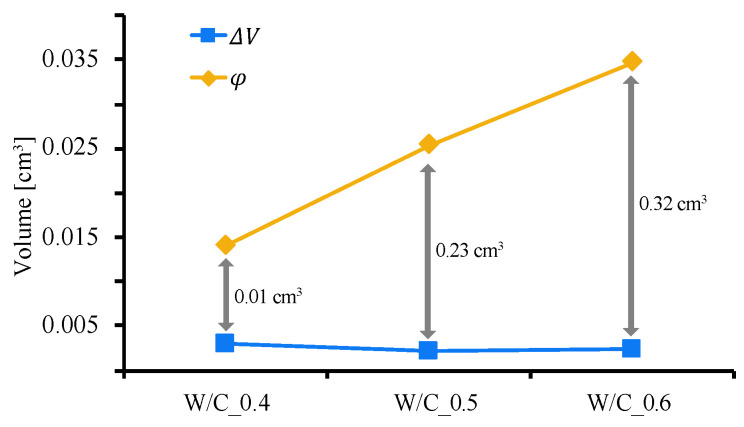
Relationship between W/C ratio, volume increase due to carbonation (ΔV), and closed pore volume (φ).

**Figure 10:**
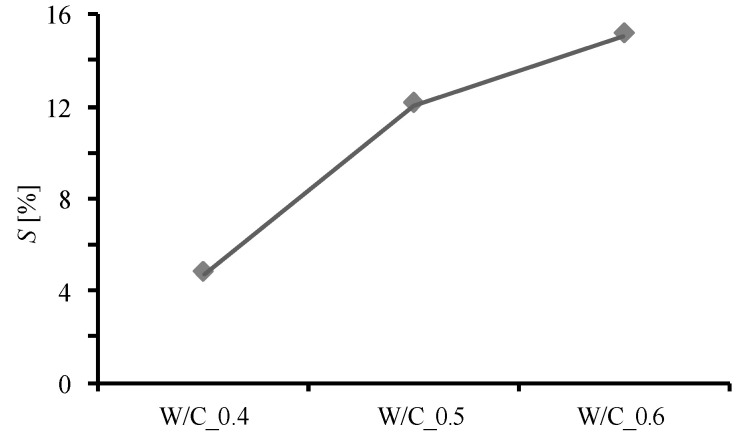
Relationship between W/C ratio and the closed pore volume increase rate per unit volume increase due to the generation of CaCO_3_ (S).

Figure 9 shows the relationship between the W/C ratio and ΔV and φ, and Figure 10 shows the relationship between the W/C ratio and S, where $V_{0\;}$ = 1 cm^3^. There is no significant difference in ΔV due to the precipitation of CaCO_3_ for each W/C ratio, but it can be seen that φ due to clogging increases as the W/C ratio increases. It is also confirmed that S increases as the W/C ratio increases. This means that even if the volume deposited by the carbonation reaction (ΔV) is the same, the void-filling effect is enhanced by creating closed pores (as shown on the right in Figure 8). This observation accordingly explains the results of this study, which indicate that water absorption is lower than the substantial precipitation volume. It is also suggested that the higher the W/C ratio, the greater the effect of the closed pores. From these facts, it is considered that when a carbonation reaction caused by CO_2_ nanobubble water is used to fill the pores in concrete, the void-filling effect is larger than the actual amount of precipitation and is higher when the W/C ratio is larger. These results indicate that in structures with a connected network pore structure, CO_2_ nanobubble has the potential to exhibit a better pore filling effect. Conversely, in the case of targeting a structure having limited pore connectivity, there is a possibility that the pore filling effect through CO_2_ nanobubbles is relatively low. In this regard, we believe that further research is needed.

## 4. Conclusions

In this study, mortar specimens with different W/C ratios were repeatedly immersed in a CO_2_ nanobubble solution following an immersion procedure to determine the effects of this treatment on the density of the specimens. The findings obtained in this study are as follows:

(1) The rate of mortar weight increase due to repeated immersion in CO_2_ nanobubble water increased as the number of immersions increased, but the appropriate number of immersions depends on the W/C ratio of the mortar.

(2) Repeated impregnation of mortar specimens with CO_2_ nanobubble water continued to decrease their densities and increase their water absorption rates, especially at W/C = 0.5 and 0.6. This is thought to be because Ca(OH)_2_ reacts completely and destroys the C-S-H gel, resulting in a net increase in surface pores.

(3) The actual porosity reduction rate was found to be larger than that derived from the calculation, and this tendency increased as the W/C ratio increased. This is considered to be due to the clogging of the pores by carbonation precipitation. It was found that the pores into which water could penetrate were smaller in the case of clogging than in the case of no clogging, indicating that the tendency of pores to clog with precipitate was stronger when the W/C ratio was larger.

## Figures and Tables

**Table 1:** Specimen parameters utilized in experiment.

Type	Water/Cement Ratio	Cement (g)	Water (g)	Fine-Aggregate (g)
W/C_0.4	0.4	114.0	45.6	225.0
W/C_0.5	0.5	100.0	50.0	225.0
W/C_0.6	0.6	89.0	53.4	225.0

**Table 2:** Chemical composition of ordinary Portland cement.

OPC	CaO	SiO_2_	Al_2_O_3_	MgO	SO_3_	Fe_2_O_3_	K_2_O	TiO_2_	LOI
(wt%)	68.11	18.43	2.85	2.37	3.03	2.17	1.10	0.12	1.82

**Table 3:** CO_2_ nanobubble water immersion procedure.

Turn Number	Process of Immersion	Method	Time
1	CO_2_ nanobubble water	Natural immersion	30 min
2	Acetone	Natural immersion	30 min
3	Dry	Vacuum desiccator	30 min
4	Acetone	Ultrasonic immersion	2 min
5	Dry	Vacuum desiccator	30 min
6	Ca(OH)_2_ saturated aqueous solution	Ultrasonic immersion	2 min
7	Acetone	Natural immersion	30 min
8	Acetone	Ultrasonic immersion	2 min
9	Dry	Vacuum desiccator	~Until next day
